# The use of the *SLC16A1* gene as a potential marker to predict race performance in Arabian horses

**DOI:** 10.1186/s12863-019-0774-4

**Published:** 2019-09-11

**Authors:** Katarzyna Ropka-Molik, Monika Stefaniuk-Szmukier, Tomasz Szmatoła, Katarzyna Piórkowska, Monika Bugno-Poniewierska

**Affiliations:** 1 Department of Animal Molecular Biology, Laboratory of Genomics, National Research Institute of Animal Production, Krakowska 1,, 32-083 Balice, Poland; 2 Department of Horse Breeding, Institute of Animal Science, University of Agriculture in Cracow, Cracow, Poland; 3 University Centre of Veterinary Medicine, University of Agriculture in Cracow, Mickiewicza 24/28, 30-059 Cracow, Poland; 4 Department of Animals Reproduction, Anatomy and Genomics, University of Agriculture in Cracow, Cracow, Poland

**Keywords:** Arabian horses, Training, Lactate, Genetic marker, Training adaptation

## Abstract

**Background:**

Arabian horses are commonly believed to be one of the oldest and the most popular horse breeds in the world, characterized by favourable stamina traits and exercise phenotypes. During intensive training, the rates of lactate production and utilization are critical to avoid muscle fatigue and a decrease in exercise performance. The key factor determining transmembrane lactate transport is the monocarboxylate transporter 1 protein coded for by the *SLC16A1* gene. The aim of the present research was to identify polymorphisms in the coding sequence and UTRs in the equine *SLC16A1* gene and to evaluate their potential association with race performance traits in Arabian horses. Based on RNA-seq data, SNPs were identified and genotyped using PCR-RFLP or PCR-HRM methods in 254 Arabian horses that competed in flat races. An association analysis between polymorphisms and racing results was performed.

**Results:**

Novel polymorphisms in the equine *SLC16A1 locus* have been identified (missense and 5’UTR variants: g.55601543C > T and g.55589063 T > G). Analysis showed a significant association between the 5’UTR polymorphism and several racing results as follows: the possibility of winning first or second place, the number of races in which horses started and total financial benefits. The analysis also showed differences in genotype distribution depending on race distance. In the studied population, the shorter distance races were only won by TT horses. The GG and TG horses took first and second places in middle- and long-distance races, and the percentage of winning heterozygotes increased from 19.5 to 27% at the middle and long distances, respectively. The p.Val432Ile (g.55601543C > T) polymorphism was not significantly related to the analysed racing results.

**Conclusion:**

Our results showed that g.55589063 T > G polymorphism affected the possibility of winning first or second place and of competing in more races. The different distribution of genotypes depending on race distance indicated the possibility of using a SNP in the *SLC16A1* gene as a marker to predict the best race distance for a horse. The presented results provide a basis for further research to validate the use of the *SLC16A1* gene as a potential marker associated with racing performance.

**Electronic supplementary material:**

The online version of this article (10.1186/s12863-019-0774-4) contains supplementary material, which is available to authorized users.

## Background

During exercise, one of the most important factor in muscle tissue is oxygen availability, which determinates carbohydrate and lactate metabolism [1]. The production and utilization of lactate in muscles is strongly related to exercise performance [1, 2]. The increased accumulation of lactate in muscle tissue, in hypoxic conditions or muscle disorders, can lead to muscle fatigue and exercise intolerance [1]. A decrease in pH and a significant increase in blood lactate concentration are only a few of the adverse effects that affect homeostasis, muscle contraction, strength and, as a result, exercise ability [2, 3]. On the other hand, lactate can be an important source of energy in working muscles [3, 4].

To maintain the proper lactate equilibrium during exercise, selection of an appropriate training intensity to enhance aerobic capacity is critical [2, 3]. Furthermore, the presence and activity of some enzymes (e.g., lactate dehydrogenase) or transporter proteins (MCT1; MCT4) strongly determine the rate of lactate metabolism. Solute carrier family 16 member 1 (also called MCT1) – SLC16A1 – transports lactate acid and protons across the cell membrane [5, 6]. Proton-linked monocarboxylate transporters allow the rapid transfer of lactate acid through the membrane, which can be used as fuel for mitochondrial respiration in muscle tissue [7]. In humans, *SLC16A1* has been established as one of the most important genes related to exercise predisposition. Mutations within the *SLC16A1 locus* are included in genetic marker tests used for the prediction of sprint or athletic performance in athletes [8–11].

Arabian horses are commonly known as endurance activity horses. However, the 2- to 5-year-olds are introduced to flat race training and compete in least one racing season before achieving maturity and undergoing endurance training. Furthermore, Arabian horse racing is a highly lucrative branch of the horse racing business. As it is for Thoroughbreds, the types of races run are part of a worldwide scheme. The 3-year-olds begin at 1400 m. The elite races are grouped into four bands, where Group 1 has the highest prestige and Listed Races the lowest, although they are still superior to handicaps or other races. To date, several studies have estimated the environmental factors affecting the genetic and phenotypic parameters for racing performance, particularly the speed of purebred Arabian racehorses [12, 13]. On the other hand, there is a lack of information describing molecular markers that potentially influence performance in Arabian racehorses.

The previous studies’ focus on global gene expression modifications in equine muscle tissues showed the increase in expression level of the *SLC16A1* gene during long-term training periods [14]. The subsequent increase in the *SLC16A1* transcript level suggested that this gene also determines exercise performance in horses. Thus, the aim of this study was to identify polymorphisms in the coding sequence and UTRs in the equine *SLC16A1* gene and to evaluate their potential association with race performance traits in Arabian horses.

## Results

### SNPs identification and genotype frequency

RNA-seq data allowed us to screen all coding sequences and UTRs of the *SLC16A1* gene. Based on 41 transcriptomes obtained from two tissues (blood and skeletal muscle) we identified two novel polymorphisms located in exon 5 (missense variant; p.Val432Ile) and in the 5’UTR. Both identified SNPs were submitted to the dbSNP database and received accession numbers (Table 1). For the 5’UTR variant genotyping, the PCR-HRM method was used, and it allowed to detect all three genotypes (Additional file 1: Figure S1).

**Table 1:** The details of methods used to genotype both identified mutations

Identified SNP	Genetic variant	Method	Primers	Details
C > T Exon 5 NM_001081791.2:g.55601543C > T ENSECAP00000019149.1:p.Val432Ile ss#3021042926	Missens variant Val/Ile	PCR-RFLP	F CAGCTTTGTTTGGGAAAGGA R CACACCGGTCTCAACCTCTT	PCR-AmpliTaq360 (Applied Biosystem); anneling temp. 57 °C PCR-RFLP with BsrDI endonuclease, alleles: C - 248 bp T – 133, 115 bp
T > G 5’UTR NM_001081791.2:g.55589063 T > G ss#3021042925	UTR variant	PCR-HRM	F TGTTGGGGGTGCTTTATTGT R GGGGGTACTTACCTCCACCA	PCR-HRM with the use KAPA HRM FAST PCR Kits (Kapa Biosystem) on QuantStudio 7 Flex (Applied Biosystem) – 323 bp

In the case of the 5’UTR (g.55589063 T > G) polymorphism, genotyping performed for 254 horses showed a low frequency of allele G (minor allele frequency - 0.11). Most of the analysed horses had a TT genotype (79.5%) and heterozygotes accounted for 18.5%. The genotyping of the p.Val432Ile variant (g.55601543C > T) showed the predominance of the C allele (0.80) and CC genotype (59.8%). Heterozygous horses accounted for 37%, while homozygous TT horses accounted for 3.2% of the tested population. The g.55601543C > T polymorphism significantly deviated from Hardy-Weinberg Equilibrium (Table 2). Moreover, the haplotype analysis shows the lack of linkage disequilibrium between detected SNPs – the r^2^ value was 0.044.

**Table 2:** Genotypic and allelic frequencies of *SLC16A1* genes polymorphisms

	Genotypes			Alleles		HWE *p*-value
g.55589063 T > G	TT	TG	GG	T	G	0.2563
	79.5%	18.5%	2%	0.89	0.11	
n	202	47	5			
g.55601543C > T	CC	CT	TT	C	T	0.0054
	59.8%	37%	3.2%	0.80	0.20	
n	152	94	8			

*n* number of horses, *HWE* Hardy-Weinberg equilibrium (Chi-squared test)

### Association between SNPs and racing results

There was a significant association between the 5’UTR polymorphism (g.55589063 T > G) and race results. When analysis was performed without considering the race distance, the horses with GG genotypes won first and occupied second places more often than the other two genotype groups. For the all races, the GG horses won first place approximately 5.7-fold times more often and took second place approximately 2 times more often than TG and TT horses (Table 3). When analysis was performed for total first to third place ranked (total of placed from 1st to 3rd places), the GG horses occupied significantly higher places compared to horses with other genotypes (*p* < 0.05). However, the g.55589063 T > G polymorphism did not affect the possibility of winning or the possibility of coming in third to fifth place. Moreover, the GG horses have competed in more races (average numbers of all starts per horse) and have earned higher total amounts of money for their wins (Table 3; *p* < 0.05). When an association of SNP and the possibility of the winning was performed with exclusion of the effect of the number of starts (ratio between number of winning at 1st to 3rd places and average number of races), the results indicated lack of the significant differences between genotypes (Tables 3 and 4). For all races, horses with GG genotype finished the competitions at 1st to 3rd places about 1.4 times more often compared to GT and TT genotypes, but observed differences were not significant (Table 3). Furthermore, at middle and long distances the ratio values for all genotypes were similar (Table 4).

**Table 3:** Impact of detected single nucleotide polymorphisms on racing results of all analyzed Arabian horses estimated for whole race records without division into individual distances

Race results	g.55589063 T > G				g.55601543C > T		
		Mean	SE	pval		Mean	SE
1st place winner^a^	GG	4.40	1.80	a	CC	0.88	0.12
	TG	0.74	0.20	b	CT	0.86	0.11
	TT	0.75	0.07	b	TT	0.87	0.39
2nd place	GG	2.00	1.22	a	CC	0.94	0.12
	TG	0.90	0.18	b	CT	0.89	0.13
	TT	0.85	0.09	ab	TT	0.37	0.26
3rd place	GG	1.60	0.70		CC	1.08	0.11
	TG	0.91	0.16		CT	0.94	0.11
	TT	1.02	0.09		TT	0.50	0.18
Total of placed from 1st to 3rd places	GG	8.00	2.17	a	CC	2.90	0.8
	TG	2.50	0.45	b	CT	2.59	0.25
	TT	2.66	0.28	b	TT	1.75	0.67
Average numbers of all starts per horse	GG	15.00	3.11	a	CC	7.29	0.41
	TG	6.74	0.55	b	CT	7.07	0.49
	TT	6.97	0.35	b	TT	5.12	1.14
Ratio - placed (1st to 3nd) to average number of starts	GG	0.53	0.11		CC	0.40	0.01
	TG	0.37	0.01		CT	0.36	0.02
	TT	0.38	0.02		TT	0.34	0.01
Total wins showed in money ($)	GG	5038.0	1471.3	a	CC	2052.4	234.1
	TG	2704.0	949.8	ab	CT	2164.1	484.5
	TT	1869.1	188.6	b	TT	1534.2	863.5

The values were presented as means with different letters differ significantly - a, b = pval< 0.05; SE – standard error

^a^ The winning classification was consistent with International Federation of Arabian Horse Racing Authorities and International Federation of Horseracing Authorities outlines

**Table 4:** Association of g.55589063 T > polymorphism on racing results only for Arabian horses winning at two different distances (middle and long distance)

Race results	middle distance (1600 m – 2000 m)				long distance (2200 m – 3000 m)		
		Mean	SE	pval	Mean	SE	pval
1st place winner^a^	GG	7.50	0.70	a	9.00	0.70	a
	TG	3.00	1.60	b	3.66	1.96	b
	TT	3.05	1.98	b	2.41	0.87	b
2nd place^a^	GG	4.00	1.22	a	4.00	0.12	
	TG	2.00	0.18	b	3.33	0.13	
	TT	2.22	0.09	ab	2.05	0.26	
3rd place^a^	GG	3.00	0.70		3.00	0.11	
	TG	1.88	0.16		2.33	0.11	
	TT	1.67	0.09		1.70	0.18	
Total of placed from 1st to 3rd places	GG	15.00	0.8	a	15.00		a
	TG	6.11	0.25	b	9.33		b
	TT	5.25	0.67	b	6.17		b
Average numbers of starts per horse	GG	31.00	3.11	a	25.00	0.41	a
	TG	10.44	0.55	b	14.00	0.49	b
	TT	10.72	0.35	b	10.23	1.14	b
Ratio - placed (1st to 3nd) to average number of starts	GG	0.48	0.03		0.60	0.02	
	TG	0.58	0.02		0.66	0.03	
	TT	0.48	0.02		0.60	0.01	
Total wins showed in money ($)	GG	8071.4	1471.3		16,934.7	234.1	
	TG	7085	949.8		8070.0	484.5	
	TT	3463.6	188.6		5556.4	863.5	

The values were presented as means with different letters differ significantly - a, b = pval< 0.05; SE – standard error

^a^The winning classification was consistent with International Federation of Arabian Horse Racing Authorities and International Federation of Horseracing Authorities outlines

**Fig. 1:**
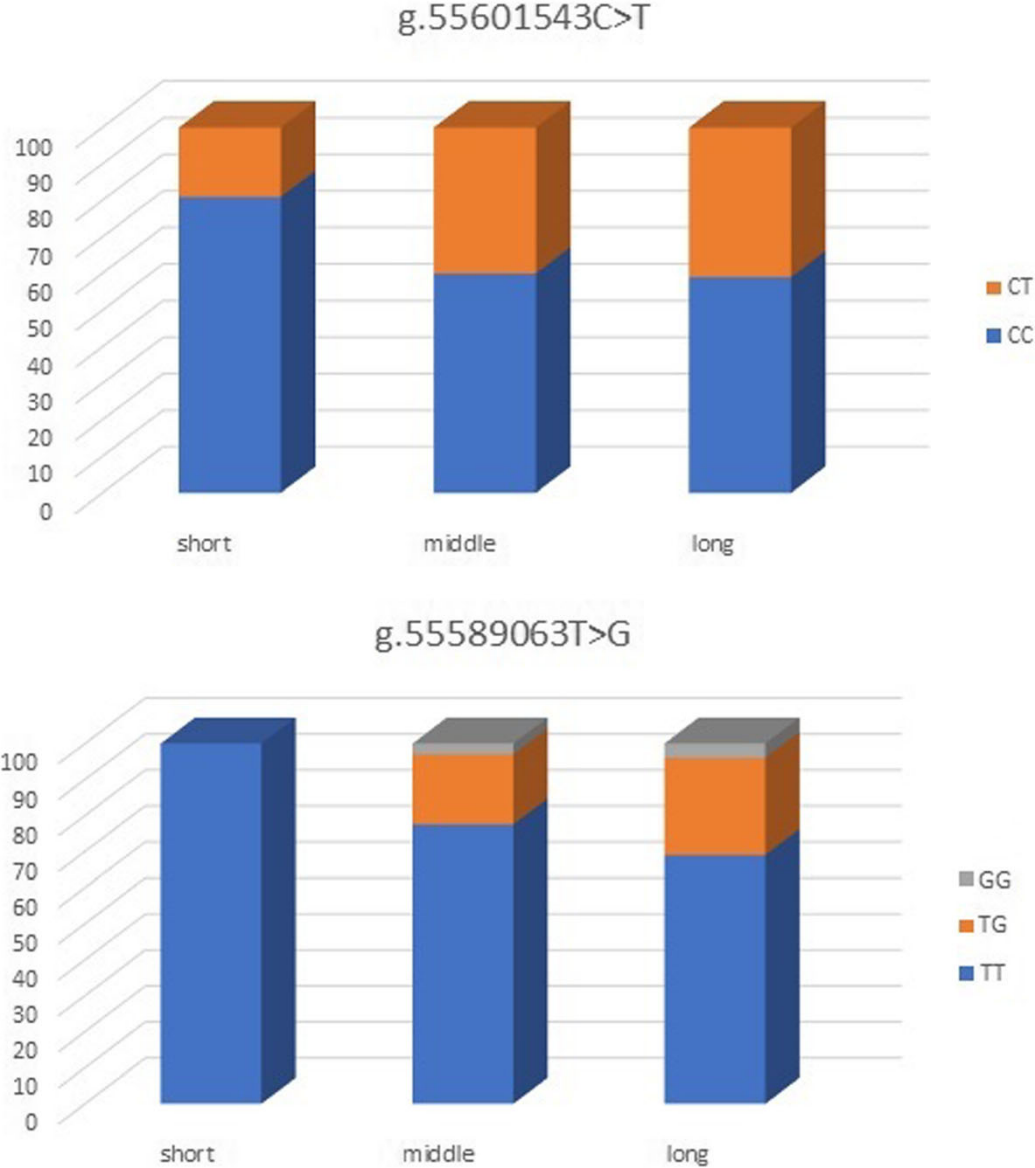
The percent genotypes distribution of horses winning races depending on race distance (the number of horses with a given genotype which win at different distances)

The analysis of genotype frequencies in each distance, showed that GG horses are not predisposed to win short-distance races but took first and second places in middle- and long-distance races, in a pattern similar to that found in TG horses. The percentage of winning horses with TG genotypes increased from 19.5 to 27% moving from middle- to long-distance races. At the 1400-m distance, the first and second places were only won by TT horses (Fig. 1). The association analysis performed for middle and long distances separately strongly confirmed the effect of g.55589063 T > G on racing results obtained for whole population (without considering the length of the race). In both distances, GG horses won significantly higher number of races and competed in the highest number of starts (*p* < 0.05) (Table 4).

Results showed that the p.Val432Ile (g.55601543C > T) polymorphism was not significantly associated with the analysed racing results (Table 3). The genotype frequency analysis showed that the number of heterozygous horses significantly increased in group of horses winning at middle and long distances compared to short races (increase from 19% at short distances to 40 and 41% at middle (*p* < 0.00000571) and long distances (*p* < 0.00001303), respectively). Horses with the TT genotype did not win first or second place at any distance (Fig. 1).

## Discussion

In horses, previous reports have shown that performance heritability for flat gallop racing can vary from 0.15 to 0.55 [14, 15]. Such high heritability indicates significant genetic background of exercise phenotypes and the strong possibility of its improving by genetic selection. In Thoroughbred horses (TB), several reports confirmed that *MSTN* gene is a major genetic factor affecting race distance aptitude and race performance [16–18]. In 2015, the GWAS approach was used to scan selective sweeps associated with selection toward genetic improvement in Thoroughbreds [19]. Authors detected regions of the genome which were under strong selection pressure and can be a background of performance traits in TB horses. In turn, Ricard et al., [20] applied the GWAS method to identify the mutations related to endurance racing performance in a French population of Arabians and crossed Arabians horses. According to global association analysis of SNPs and racing results (total race distance, average race speed, and finishing status) authors selected five quantitative trait loci (QTL) related to endurance racing performance. Two genes were selected: *SORCS3* on chromosome 1 and *SLC39A12* on chromosome 29 as the strongest and the most significant related with analyzed traits. The promising results obtained for Thoroughbred horses tend to conduct research focused on identification of genetic background of performance traits also in Arabian horses, which can lead to establishment genetic marker.

Muscle tissue accounts for the largest share of a horse’s weight and is one of the most important body components. Under anaerobic conditions, higher amount of produced lactic acid is used for energy (ATP) synthesis. During maximal exercise, the produced lactic acid can be used as a source of lactate and protons (H^+^), which accumulate in muscles, leading to decreased pH and muscle acidosis [2]. Such exercise-induced metabolic acidosis can be one of the factors leading to muscle fatigue and a decrease in exercise performance.

The key to avoiding such accumulation of lactic acid in muscle tissue is the speed and efficient mechanisms related to lactic acid transport across the sarcolemma. As an important metabolic intermediate, the lactate acid can be transferred rapidly between different cells/ fiber types within a muscle, between different muscles or between muscle and blood [5]. It has been established that two proteins belonging to monocarboxylate transporter family (*MCT1; MCT4*) are strongly involved in lactate transport and accumulation and as a result are related to changes in intercellular pH [3]. SLC16A1 (MCT1) is mainly expressed in Type I fibres (slow-twitch, oxidative), while SLC16A3 (MCT4) is in higher abundance in Type IIB fibres (glycolytic) [1, 5, 21].

Our previous study showed that *SLC16A1* gene expression is up-regulated during training regimens preparing for flat racing. In Arabian horses’ muscle tissues, the gradual increase of the *SLC16A1* transcript level was detected starting from untrained horses up to horses at the top of their training form [22]. The increases in both training intensity and *SLC16A1* gene expression confirmed that this protein can play one of an important role in the adaptation of muscles to exercise. The ratio of lactate transport across cell membranes and utilization in cells to its accumulation is critical to maintain homeostasis and proper muscle function [23]. This is especially important for horses in which the high-intensity muscular exercise results in acidification of muscles and blood [24]. Furthermore, our association study showed that there was a significant relation between g.55589063 T > G SNP located in the 5’UTR and the selected racing results. It has been established that 5′ untranslated region play a critical role during translation initiation in both cap-dependent or cap-independent manner [25]. The 5’UTR is strongly related to helicase-mediated remodelling of RNA structures, thus the detected g.55589063 T > G variant might affect *SLC16A1* protein content as well as gene expression level [26]. In our study, GG horses won first and occupied second places significantly more often and started in higher numbers of races than TG and TT horses. On the other hand, a very low frequency of GG horses (2% of the population tested) were detected. The obtained results confirmed also significant effect of the number of races in which horses participated on total winnings. Moreover, horses with a GG genotype competed in a highest number of races and thus had a greater probability of winning. However, their participation in more races might be a result of better predispositions to races evaluated by previous competitions.

The low frequency of the desirable TT genotype associated with elite sprint/power athletic status was also identified in human [9]. The missense polymorphism (p.Asp490Glu, rs1049434) that modifies SLC16A1 activity has been identified by several studies [8, 9]. The p.Asp490Glu polymorphism was selected as genetic marker used to evaluate predisposition for sprint/power or endurance [9, 27]. The authors found significant differences in the numbers of TT individuals in groups predisposed for endurance, sprint/power effort and control (14, 27 and 12.6%; respectively). Additionally, when the analysis was performed separately for each athlete phenotype, 19% more TT individuals were detected in elite category compared to national-level athletes in the sprint/power group. Our study showed that races over short distances (sprint) were only won by TT horses (g.55589063 T > G), while at middle and long distances, the number of TG and GG horses winning significantly increased. The observed genotype distribution might be due to a possible modification of the function of SLC16A1 via expression or translation regulation (polymorphism in 5’UTR), which can result in differences in response to exercise training.

The second analysed polymorphism changes valine to isoleucine at position 432 in the amino acid chain. This *SLC16A1* gene region encodes the tenth and final transmembrane portion of the MCT1 protein and such an amino acid substitution could lead to the partial loss of or change in the monocarboxylate transporter function. Similarly, the mutation identified in humans (p.Asp490Glu) results in an amino acid substitution in the final non-membrane (topological) domain, which modifies MCT1 protein activity, decreasing lactate transport and increasing the blood lactate concentration [28]. In our study, we did not identify any statistically significant effects of the p.Val432Ile mutation on race results, but some trends were observed. The identified missense variant had a negative effect on the number of races in which horses participated and the possibilities of winning second and third places (homozygous TT horses compared to wild type, CC). Homozygous TT horses did not win first or second places at any distance. Thus, the very low frequencies of TT horses and the obtained racing results suggest that the p.Val432Ile mutation may also modify MCT1 function, but by reducing its activity.

## Conclusion

The present research allowed us to detect two new polymorphisms in the *SLC16A1 locus* that can affect the biological function of the lactate acid transporter. The previous transcriptomic analysis showed that the *SLC16A1* gene is up-regulated during a training regimen, which may mean that it is related to the body’s adaptation to intense exercise. The SNP located in the 5’UTR affected the possibility of achieving first and second places and of competing in more races. The presented results provide a basis for further research to validate the use of the *SLC16A1* gene as a potential marker associated with racing performance.

## Methods

### Animals, samples and racing results

The analysis was performed on 254 pure Arabian horses (185 mares and 69 stallions). The analyzed Arabian horses were that offspring of 95 stallions (an average 2.67 individual per sire) and 208 mares (an average 1.22 individual per dam). Additionally, based on SNP microarray data (from the other project on the same population) the inbreeding coefficient - FIS (Excess homozygosity-based inbreeding estimate) was calculated and was 0.049472, which indicated on low relatedness (however, the FIS value above 0 might denote on slight increasing the relationships between animals in analysed population). All horses are registered as pure-breed in Polish Arabian Stud Book (PASB) being under World Arabian Horse Organization. Horses were 3 to 5 years old and had all taken part in flat races. Horses participated in flat races at distances ranging from 1400 to 3000 m. For each animal, several racing results were collected: the number of wins and rate of placing first, second, third, fourth and fifth, the number of races run and the distances of the races in which each horse participated. The total money winnings and dam line effect were also taken into consideration. All horses which participated in flat-races were genotyped regardless of their racing results, thus analysed population included horses with excellent or good results and horses which participated in races, but they did not win any race/money. The analyzed Arabian horses competing in flat races under the management of Horse Racing Authority in Poland affiliated by International Federation of Arabian Horse Racing Authorities and International Federation of Horseracing Authorities. The winning classification was consistent with outlines of the above societies.

Whole blood or hair follicles were collected from the horses. The protocol was approved by the Animal Care and Use Committee of the Institute of Pharmacology, Polish Academy of Sciences in Kraków (no.1173/2015).

### SNP identification and genotyping

All SNPs within *SLC16A1* were detected based on RNA-seq data previously obtained from Arabian horses according to EquCab2.0 reference [22, 29]. Then, the fast and less cost-effective PCR-RFLP method was designed to identify polymorphisms in exon 5 (ss#3021042926), and PCR-HRM was used to identify mutation in the 5’UTR (ss#3021042925). The details of the methods used are presented in Table 1. For 48 randomly selected samples, the both amplicons were sequenced using Sanger sequencing to confirm the results obtained. The sequencing was performed using BigDye Terminator v3.1 Cycle Sequencing Kit (Applied Biosysytems, Thermo Fisher Scientific), PCR products were purified using BigDye XTerminator Purification Kit (Applied Biosysytems) and next sequenced on 3500xl Genetic Analyzer (Applied Biosysytems). DNA samples from blood and hair follicles were isolated with the use of Sherlock AX DNA Isolation kit (A&A Biotechnology, Gdynia, Poland), according to protocol. Both polymorphisms were genotyped for all horses.

### Statistical analysis

The races in which the analysed horses participated and won first place were assigned to one of three distance groups:

- distance 1 – short – 1400 m
- distance 2 – middle – 1600 m – 2000 m
- distance 3 – long – 2200 m – 3000 m

The differences in genotype frequencies between distance groups were calculated with the use of a chi-square test (R Package). The normality of racing data distribution was tested using Shapiro-Wilk test. The association between the both identified mutations and the racing results were estimated using the GLM procedure. The GLM model in the most expanded form included all the factors of interest:

$$ {\mathrm{Y}}_{\mathrm{i}\mathrm{jklm}}=\upmu +{\mathrm{AGE}}_{\mathrm{i}}+{\mathrm{GEN}}_{\mathrm{j}}+{\mathrm{SEX}}_{\mathrm{k}}+{\mathrm{MATL}}_{\mathrm{l}}+{\mathrm{RSD}}_{\mathrm{m}}+{\mathrm{e}}_{\mathrm{i}\mathrm{jklm}} $$

where:

Y_ijklm_ – the trait measured (1st place winner; 2nd place; 2nd place; total of placed from 1st to 3rd places; average numbers of all starts per horse; ratio- placed (1st to 3nd) to average number of starts; total wins showed in money).

μ – the overall mean for the trait measured,

AGE_i_ - the fixed effect of i horse age (from 3.5 year to 5 year),

GEN_j_ – the fixed effect of j genotype group of *SLC16A1* gene (TT, TG, GG for g.55589063 T > G or CC, CT TT for g.55601543C > T),

SEX_k_ – the fixed effect of k sex,

MATL_j_ – the fixed effect of l maternal line (from 1 to 23)*,

RSD_m_ – the random d effect of m rider or sire or dam (each of the effects of the rider, sire or dam was included in the model separately),

e_ijklm_ – random error.

Next, the factors with *p*-values more than 0.05 were removed and final model was:

$$ {\mathrm{Y}}_{\mathrm{ijklm}}=\upmu +{\mathrm{GEN}}_{\mathrm{j}}+{\mathrm{SEX}}_{\mathrm{k}}+{\mathrm{MATL}}_{\mathrm{l}}+{\mathrm{e}}_{\mathrm{j}\mathrm{kl}} $$

where:

Y_ijklm_ – the trait measured,

μ – the overall mean for the trait,

GEN_j_ – the fixed effect of j genotype group of *SLC16A1* gene (TT, TG, GG for g.55589063 T > G or CC, CT TT for g.55601543C > T),

SEX_k_ – the fixed effect of k sex (significant for number of races starts per horse).

MATL_j_ – the fixed effect of l maternal line (from 1 to 23)*,

e_jkl_ – random error.

*The maternal line classification was done based on the pedigrees of the horses (http://www.janow.arabians.pl/pl/rodowod-form.php). First, all horses were divided into groups according to the matrilineal founders. Second, the sub lines were extracted where the founder progeny had at least 6 generations and had established its own line with living and approved successors. The detailed information about dam lines is presented in Additional file 2: Table S1.

As a post-hoc test we used Duncan’s new multiple range test. The analysis was performed for whole population and separately for horses winning at middle and long distances.

The linkage disequilibrium between SNPs were calculated using R Bioconductor package – Chopsticks v1.46.0 [30].

## Additional files

Additional file 1: **Figure S1.** The derived melt curves specific for each detected genotype obtained by PCR-HRM method. (JPG 133 kb)

Additional file 2: **Table S1.** The dam lines classification with matrilineal founders and number of generations. (DOC 62 kb)

## Data Availability

The original data of the paper are available upon request from the corresponding author.

## Abbreviations

SNP: Single nucleotide polymorphism
TB: Thoroughbred horses
UTR: Untranslated region
