# Supplementary figures and images for: The use of the SLC16A1 gene as a potential marker to predict race performance in Arabian horses
Source: BMC Genet. 2019 Sep 11;20:73. doi: 10.1186/s12863-019-0774-4 (PMC6740031; doi:10.1186/s12863-019-0774-4)

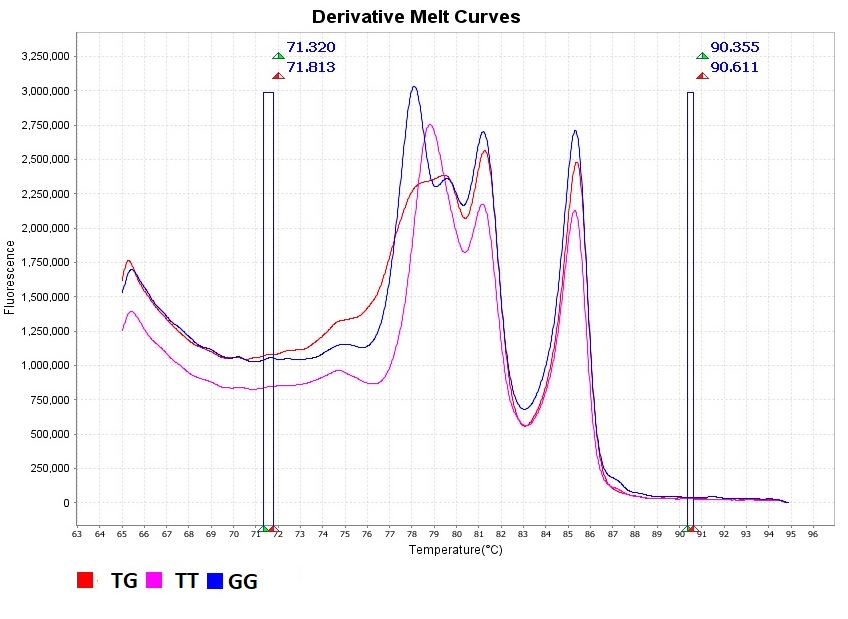

Supplement: Supplementary file 1 — Figure S1. The derived melt curves specific for each detected genotype obtained by PCR-HRM method. (JPG 133 kb) [file 12863_2019_774_MOESM1_ESM.jpg]
