# Supplementary material for: The use of the SLC16A1 gene as a potential marker to predict race performance in Arabian horses
Source: BMC Genet. 2019 Sep 11;20:73. doi: 10.1186/s12863-019-0774-4 (PMC6740031; doi:10.1186/s12863-019-0774-4)
Supplement: Supplementary file 2 — Table S1. The dam lines classification with matrilineal founders and number of generations. (DOC 62 kb) [file 12863_2019_774_MOESM2_ESM.doc]

Additional file, Table S1. The dam lines classification with matrilineal founders and number of generations.

| **Source Stud** | **Matrilinear line founder in Poland** | **The founder of subline** | | **Number of generations within line (subline)** | **Line’s abbreviation** |
| --- | --- | --- | --- | --- | --- |
| Tiaret (France) | Cherifa or.ar. | Elka (PL) 1960 | | 16 (5) | CHE |
| Babolna (Hungary) | Scherife or.ar. 1896 | Algeria 1971 | | 11 (5) | AS |
| Słwuta (Poland) | Szweykowska 1803 | Elstera 1913 | | 20 (12) | SE |
|  |  | Kalina 1909 | | 20 (20) | SK |
|  | Wołoszka 1810 | Karima 1924 | | 22 (11) | WK |
|  |  | Niespodzianka 1931 | | 22 (8) | WN |
|  | Milordka 1816 | Delfina 1825 | | 23 (22) | MDE |
|  |  | Zaira 1826 | Siglavy Bagdady 1908 | 23 (13) | MZJ |
|  |  |  | 238 Amurath-25 1901 | 23 (12) | MZF |
|  | Ukrainka 1815 | Dyska 1950 | |  | UD |
|  |  | Fatma 1961 | |  | UF |
| Crabbet Park (Great Britain) | Selma or.ar | Sardhana 1924 | | 12 (9) | SZ |
| Biała Cerkiew | Szamrajówka 1810 | Pierzga 1964 | | 19 (7) | SPI |
|  |  | Pentoda 1970 | | 19 (7) | SPE |
| Jarczowce | Gazella or.ar. ~1840, | Łania * | Fryga II 1924  Elegantka 1923  Jaskółka II 1928  Taraszcza 1937 | 19 (11) | GLF |
|  |  |  |  | 19 (10) | GLE |
|  |  |  |  | 19 (9) | GLJ |
|  |  |  |  | 19 (8) | GLT |
|  |  | Gazella II * | | 19 (17) | GGG |
|  | Mlecha or.ar. 1845 | Rozmaita I |  | 15 (20) | MRS |
|  |  | Mlecha-Elazus | Iwonka III 1936 | 17 (10) | MMI |
|  |  |  | Mabrucha 1928 | 17 (8) | MMM |
|  |  |  | Safta 1929 | 17 (9) | MMP |
|  | Sahara or.ar 1845 | Daribba 1950 | | 18 (7) | SDD |
|  |  | Epigona 1953 | | 18 (5) | SEE |
